# CHD4 mediates proliferation and migration of non-small cell lung cancer via the RhoA/ROCK pathway by regulating PHF5A

**DOI:** 10.1186/s12885-020-06762-z

**Published:** 2020-03-30

**Authors:** Nuo Xu, Fanglei Liu, Shengdi Wu, Maosong Ye, Haiyan Ge, Meiling Zhang, Yuanlin Song, Lin Tong, Jian Zhou, Chunxue Bai

**Affiliations:** 1 Department of Pulmonary Medicine, Zhongshan Hospital, Fudan University, 180 Fenglin Road, Shanghai, 200032 China; 2 Department of Pulmonary Medicine, Shanghai East Hospital, Tongji University, Shanghai, 200120 China; 3 Department of Gastroenterology and Hepatology, Zhongshan Hospital, Fudan University, Shanghai, 200032 China; 4 Department of Pulmonary Medicine, Huadong Hospital, Shanghai, 200040 China

**Keywords:** CHD4, metastasis, non-small cell lung cancer, PHF5A, proliferation

## Abstract

**Background:**

Chromodomain helicase DNA-binding protein 4 (CHD4) has been shown to contribute to DNA repair and cell cycle promotion; however, its roles in cancer initiation and progression remain largely unknown. This study aimed to demonstrate the role of CHD4 in the development of non-small cell lung cancer (NSCLC) and determine the potential mechanisms of action.

**Methods:**

By using immunohistochemistry, the expression levels were evaluated in both cancer and non-cancerous tissues. Subsequently, CHD4 knockdown and overexpression strategies were employed to investigate the effects of CHD4 on cell proliferation, migration, along with the growth and formation of tumors in a xenografts mouse model. The protein expression levels of CHD4, PHF5A and ROCK/RhoA markers were determined by Western blot analysis.

**Results:**

Compared with non-cancerous tissues, CHD4 was overexpressed in cancer tissues and CHD4 expression levels were closely related to clinical parameters of NSCLC patients. In H292 and PC-9 cell lines, CHD4 overexpression could promote the proliferative and migratory potential of NSCLC cells. Furthermore, down-regulation of CHD4 could reduce the proliferative and migratory ability in A549 and H1299 cell lines. Meanwhile, knockdown of CHD4 could decrease the tumorigenicity in nude mice. Finally, we demonstrated that one of the mechanisms underlying the promotive effect of CHD4 on NSCLC proliferation and migration may be through its interaction with PHD finger protein 5A (PHF5A) and subsequent activation of the RhoA/ROCK signaling pathway.

**Conclusions:**

CHD4, which is highly expressed in cancer tissue, could be an independent prognostic factor for NSCLC patients. CHD4 plays an important role in regulating the proliferative and migratory abilities of NSCLC via likely the RhoA/ROCK pathway by regulating PHF5A.

## Background

Non-small cell lung cancer (NSCLC) is the most common type of lung cancer. Chemotherapy and radiotherapy, have reached a therapeutic plateau. Immune therapy and targeted therapies are only effective in the small subset of NSCLC patients [1–4]. The identification and characterization of genes that play important roles in cancer development and progression could lead to new approaches for its diagnosis and treatment.

Chromodomain helicase DNA-binding protein 4 (CHD4), a chromatin remodeling factor, is an integral component of the nucleosome remodeling deacetylase (NuRD) complex, which is unique in combining chromatin remodeling activity with histone deacetylase and demethylase functions involved in transcriptional repression [5]. Sims et al. demonstrated that depletion of the catalytic ATPase subunit of CHD4 in cells with a dampened DNA damage response (DDR) resulted in a slow-growth phenotype characterized by a delayed progression through S phase [6]. Recently, Wang et al. has found that TRPS1 and CHD4/NuRD formed complex and play a role in cancer cell migration and invasion by repressing TP63 expression in breast and kidney cancer cells [7]. Increased CHD4 expression has also been detected in ovarian and oral cancer cells [8, 9]. In a study in uterine serous carcinoma, somatic copy-number variations indicated amplification of CHD4 in 7 of 25 tumors (28%) [10]. However, in a recent study, CHD4 was found to be one of the tumor suppressing TF (transcriptional factor) in lung cancer. It is reported that median OS (overall survival) of patients with high levels of these genes was significantly longer than that of cases with low levels of the genes [11]. Thus, the role of CHD4 in NSCLC remains quite obscure. In this study, we investigated the role of CHD4 in the growth and migration of NSCLC using suppression and overexpression strategies in vitro and in vivo*.*

## Methods

### Patients and tumor samples

Between January 2005 and February 2009, a total of 242 patients with histologically confirmed NSCLC were consecutively treated for NSCLC at Zhongshan Hospital of Fudan University. Specimens of both tumor and adjacent non-tumor tissue were collected at the operation. Pathologists were helping to ensure correct sampling of tissues from the tumor, adjacent non-tumor lung tissues(3–5 cm from the tumor), without adversely affecting the participant. The pathologists classified the samples as tumor and corresponding adjacent non-tumor lung tissues. The TNM status was determined according to the 8th edition staging system for NSCLC [12]. Patients with R1/R2 resection, survival < 30 days after surgery, who died due to other causes or were lost to follow-up were excluded from the study. In total, 96 patients were excluded from the analysis, including 4 patients who had previously received radiotherapy and/or chemotherapy, 51 patients with poor quality and/or quantity of tissue samples, 19 patients with incomplete clinical data, and 22 patients who died of other causes. Finally, a total of 146 patients who underwent curative surgical resection were included in this study. Of these, 73 patients were alive at the end of the follow-up, and 73 patients died from lung cancer. The clinicopathological data for each patient, including sex, age, tumor stage, nodal status, TNM stage, histological grade and overall survival, were obtained retrospectively from the clinical records and pathological reports. The pathologists who performed the immunohistochemical assessment of CHD4 were blinded to the patients’ histopathologic and follow-up data.

The survival time was defined as the duration from the date of diagnosis to the date of death or the end of the follow-up.

### Antibodies

The following antibodies were used in this study: CHD4 (ab72418, Abcam, polyclonal, dilution: 1:1000); PHF5A (ab103075; Abcam, polyclonal, dilution: 1:1000); myosin (MY-21, M4401, Sigma, monoclonal, dilution: 1:200); phospho–myosin (sc-12,896, Santa Cruz, monoclonal, dilution:1:200); ROCK (#4035S, Cell Signaling Technology, monoclonal, dilution:1:1000); RhoA (#2117, Cell Signaling Technology, monoclonal, dilution:1:1000). E-cadherin (#3195, Cell signaling Technology, monoclonal, dilution:1:1000); ERK (#4348S,Cell Signaling Technology, monoclonal, dilution:1:1000) and p-ERK (#4370, Cell Signaling Technology, monoclonal, dilution:1:2000).

### Western blot analysis

The protein concentration was measured by the Bradford assay. Cell lysates were separated by SDS-PAGE and transferred to poly vinylidene difluoride membranes. The membranes were blocked with 5% non-fat milk in TBST and incubated with specific primary antibodies. The band intensities were measured by using SuperSignal West Pico chemi-luminescent substrate (Thermo Scientific) followed by exposure to X-ray film. After that, quantification was performed using the Image J software.

### Immunohistochemical techniques

Paraffin-embedded tissue blocks were sectioned (3 μm) for immunohistochemical staining. Sections were immersed in xylene, alcohol and washed with PBS for three times after each immersion. After protein denature, using microwave and non-specific biding blocking with normal goat serum for 20 min at room tempreture. The sections were then incubated with rabbit polyclonal antibody against CHD4 (ab72418, Abcam) with 1:100 dilution for experimental slides overnight at 4 °C. The secondary antibody was Bond Polymer Refine Detection (DS9800). The sections were incubated with 3,3′-Diaminobenzidine (DAB) and hematoxylin staining.

CHD4 expression was observed in the cell cytoplasm and nucleus. Staining was assessed in five high-powered fields, and three sections per specimen were assessed. The percentage of the area that was positively stained was categorized into the following four groups: < 25% of the tumor cells stained, 0; 25–50% stained, 1; 50–75% stained, 2; and > 75% stained, 3. The staining score was categorized into four groups as follows: negative, 0; weak, 1; moderate, 2; and intense, 3. The labeling score was determined by multiplying the stained area score by the intensity score, with potential scores of 0, 1, 2, 3, 4, 6 and 9. Then, the labeling score was categorized into two groups: weak/negative staining (score < 4) and strong staining (score ≥ 4) [13]. Among the three tissue sections, the highest labeling score was entered for the statistical analyses. The pathologists were blinded to the patients’ follow-up data.

### RNA interference

The individual small interfering RNAs (siRNAs) were obtained from Shenggong, Inc., Shanghai, China. The annotations and sequences of the siRNAs were as follows (sense strands):,5′-CGGGUAUUGAAUGGUUACUTT-3′; and control siRNA, 5′-UUCUCCGAACGUGUCACGUTTACGUGACACGUUCGGAGAATT-3′. The siRNA transfections were performed with 100 nM siRNA duplexes using Lipofectamine RNAiMAX (Invitrogen, USA). The cells were transfected with siRNAs 24 h after plating. The samples were harvested 72 h after transfection initiation, unless stated otherwise.

### Tumor cell migration assays

Assays to measure tumor cell migration were performed in a modified Boyden chamber (Transwell, Corning Costar, MA, USA) containing a gelatin-coated polycarbonate membrane filter (8-μm pore size). Cells were seeded at a density of 40,000 cells per well, and the wells were washed with D-PBS after 24 h. The degree of tumor cell migration and was evaluated according to previous protocols [14]. Cell counting was performed following Coomassie blue staining, and the cells were subsequently visualized under a microscope (Leica, Inc., Solms, Germany).

### Co-immunoprecipitation (co-IP)

Cells were washed with phosphate-buffered saline (PBS) and lysed with ice-cold NETN buffer containing protease inhibitor. The lysates were centrifuged to remove cell debris. The samples were incubated with antibody overnight at 4 °C and then the whole-cell extracts were precleared with pre-washed protein A/G-conjugated agarose beads for 2 h at 4 °C. Agarose beads were then washed with NETN buffer and immunoprecipitates were collected by boiling beads in 800 μ1*SDS sample buffer for 10 min. Finally, the supernatant was subjected to SDS-PAGE and subsequent western blotting analysis.

### Flow cytometry

The cells were harvested and resuspended in 200 μl of propidium iodide (PI) buffer. The samples were incubated for 30 min at 37 °C before analysis. Cell cycle analysis was performed using a flow cytometer (FACSCalibur; BD Biosciences).

### Real-time PCR

Real-time PCR was performed as described previously [15], with the following primer sequences: CHD4, (sense) 5′-CAAGAAGCCTAAACCCAAGAAA-3′ and (antisense) 5′-CCACATCTAAGTCATCATCCTCAC-3′; and PHF5A, (sense) 5′-GCTTGAGGAACTGACTGTGAAG-3′ and (antisense) 5′-AAACGGGAAATGCCTACAT-3′.

### Animal experiments

A total of 20 Four- to five-week-old male BALB/CA nude mice (purchased from the Shanghai Institute of Material Medicine, Chinese Academy of Science, Shanghai, China) were maintained under specific pathogen-free (SPF) conditions. CHD4-down-regulated A549 or A549-NC cells (5 × 10^6^ per mouse) were injected subcutaneously into the right lower flanks of the nude mice (*n* = 6 per group). Five weeks later, the mice were sacrificed by cervical dislocation and the tumors were removed and measured for analysis.

### ChIP-qPCR and gene ontology (GO) functional analysis

Cells were treated to create protein–DNA crosslinks, and the crosslinked sheared chromatin was used for immune-precipitation with normal IgG, or PHF5A antibodies. The immuno-precipitates were washed, eluted, and de-crosslinked, followed by quantification PCR. Total RNA isolated were used to prepare cDNA libraries that were subsequently sequenced on the Illumina HiSeq2500. Raw reads were mapped to the genome with Bowtie (version 2). Peak calling was performed by MACS. Motif analysis was performed using MEME-ChIP, and Pathway enrichment analysis were identified using Kyoto Encyclopedia of Genes and Genomes (KEGG) and Gene Ontology (GO) functional analysis by blast2go software.

### Data analyses

A number of clinicopathological factors were evaluated. Fisher’s exact test was used to evaluate the associations between the clinicopathological variables of the patients and the expression of CHD4. A *P*-value of 0.05 was considered to be significant in all analyses. The clinicopathological variables and CHD4 expression were also subjected to survival analysis using the Kaplan–Meier method, and potential heterogeneity among the studies was quantified using chi-squared test. Multivariate analysis was performed with the Cox proportional hazards regression model to examine the independent prognostic effect of CHD4 on survival by adjusting for the confounding factors. Statistical analysis of the differences between the animal or cellular groups was performed with an unpaired student’s t-test., (two-tailed; *P* < 0.05 was considered significant). The results are presented as mean ± s.e.m. *, *P* < 0.05; **, *P* < 0.01; ***, *P* < 0.001. SPSS 19.0 was used to perform all statistical analyses in this study.

## Results

### Identification of CHD4 as an NSCLC-associated gene and correlation of CHD4 expression with NSCLC clinicopathological features

**Fig. 1:**
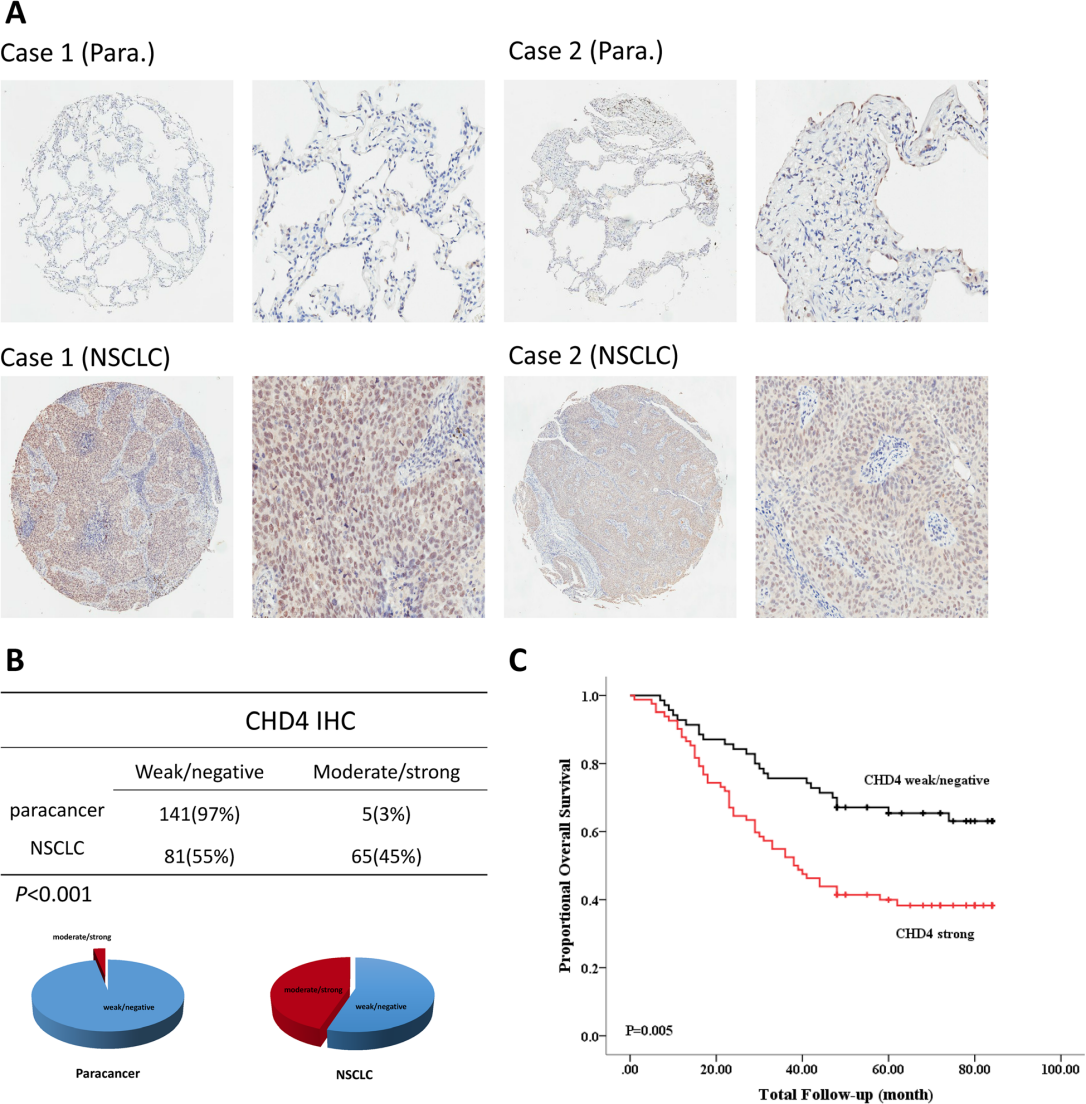
Up-regulated CHD4 was associated with a substantially poorer prognosis in NSCLC patients. **a** Representative IHC images of CHD4 in NSCLC and adjacent non-cancerous tissues. Left, magnification × 50; right, magnification × 200. **b** The IHC analysis of CHD4 in an independent set of paired NSCLC and matching non-tumor tissues; IHC signal intensities were scored as weak/negative or moderate/strong. The pie chart represented the proportions of NSCLC samples showing different intensities of IHC staining of CHD4. **c** The overall survival of 146 NSCLC patients with weak/negative or moderate/strong staining of CHD4. *P* < 0.05 was considered significant. IHC, immunohistochemistry

To discover whether CHD4 plays an important role in NSCLC, we examined CHD4 expression in 146 paired NSCLC and adjacent non-cancerous tissues (Fig. 1a). In non-cancerous tissues, 141 of 146 (97%) samples exhibited weak or negative staining, whereas 5 of 146 (3%) samples showed moderate or strong staining. By contrast, in cancer tissues, 81 of 146 (55%) samples had weak or negative CHD4 expression, whereas 65 of 146 (45%) samples had moderate or strong CHD4 expression (Fig. 1b), demonstrating that the expression of CHD4 was higher in NSCLC tumor tissues than in adjacent non-cancerous tissues.

To further determine the clinicopathological significance of CHD4 in NSCLC, we analyzed the relation between CHD4 expression levels and clinicopathological parameters(Table 1). The results showed that the expression level of CHD4 was significantly associated with TNM stage(*P* = 0.001), tumor size(*P* = 0.002) and lymph node metastasis (*P* = 0.005)(Table S1). CHD4 expression levels were also significantly associated with the overall survival of NSCLC patients(*P* = 0.005) (Fig. 1c). The stronger the CHD4 expression, the shorter the patient survival time. In addition, the multivariate analysis revealed that CHD4 could be used as an independent factor for predicting NSCLC patient prognosis (*P* = 0.024)(Table 2). Taken together, these results suggested that CHD4 overexpression is a critical factor in NSCLC development and progression.

**Table 1:** Characteristics of patients with non-small cell lung cancer

*Characteristics*	*Patients(No. = 146)*
*Age (years)*	
≤ 55	29(50.9%)
> 55	117(49.1%)
*Gender*	
Male	107(69.7%)
Female	39(30.3%)
*Smoking status*	
Yes	87(55.4%)
No	59(44.6%)
*Histology*	
Adenocarcinoma	80(67.4%)
Squamous cell carcinoma	66(32.6%)
*Differentiation*	
Well and moderately	75(53.1%)
Poorly	71(46.9%)
*TNM Stage*	
I	75(43.4%)
II	22(13.2%)
III	42(29.1%)
IV	7(14.3%)
*Tumor stage*	
T1	42(20.6%)
T2	69(42.8%)
T3	19(14.3%)
T4	16(22.3%)
*Lymph node metastasis*	
No	88(52%)
Yes	58(48%)

*Abbreviation*: *No.* number, *TNM* tumor node metastasis

*, significant

**Table 2:** Multivariable analysis for the effect of CHD4 expression on survival

*Variable*	*P value*	*Hazard ratio*	*95%CI*
*Age*	0.218	1.5	0.35–1.27
*Gender*	0.924	0.97	0.57–1.85
*Histology*	0.703	0.89	0.63–1.96
*CHD4 expression*	0.024*	1.85	0.33–0.93
*Tumor stage*	0.521	1.22	0.70–2.02
*TNM Stage*	0.003*	3.64	0.12–0.66
*Lymph node metastasis*	0.507	1.35	0.71–2.03
*Differentiation*	0.545	0.85	0.69–2.00

*Abbreviation*: *No.* number, *TNM* tumor node metastasis; 95%CI, 95% confidence interval. *, significant

### Down-regulation of CHD4 inhibits NSCLC cell migration and proliferation in vitro

**Fig. 2:**
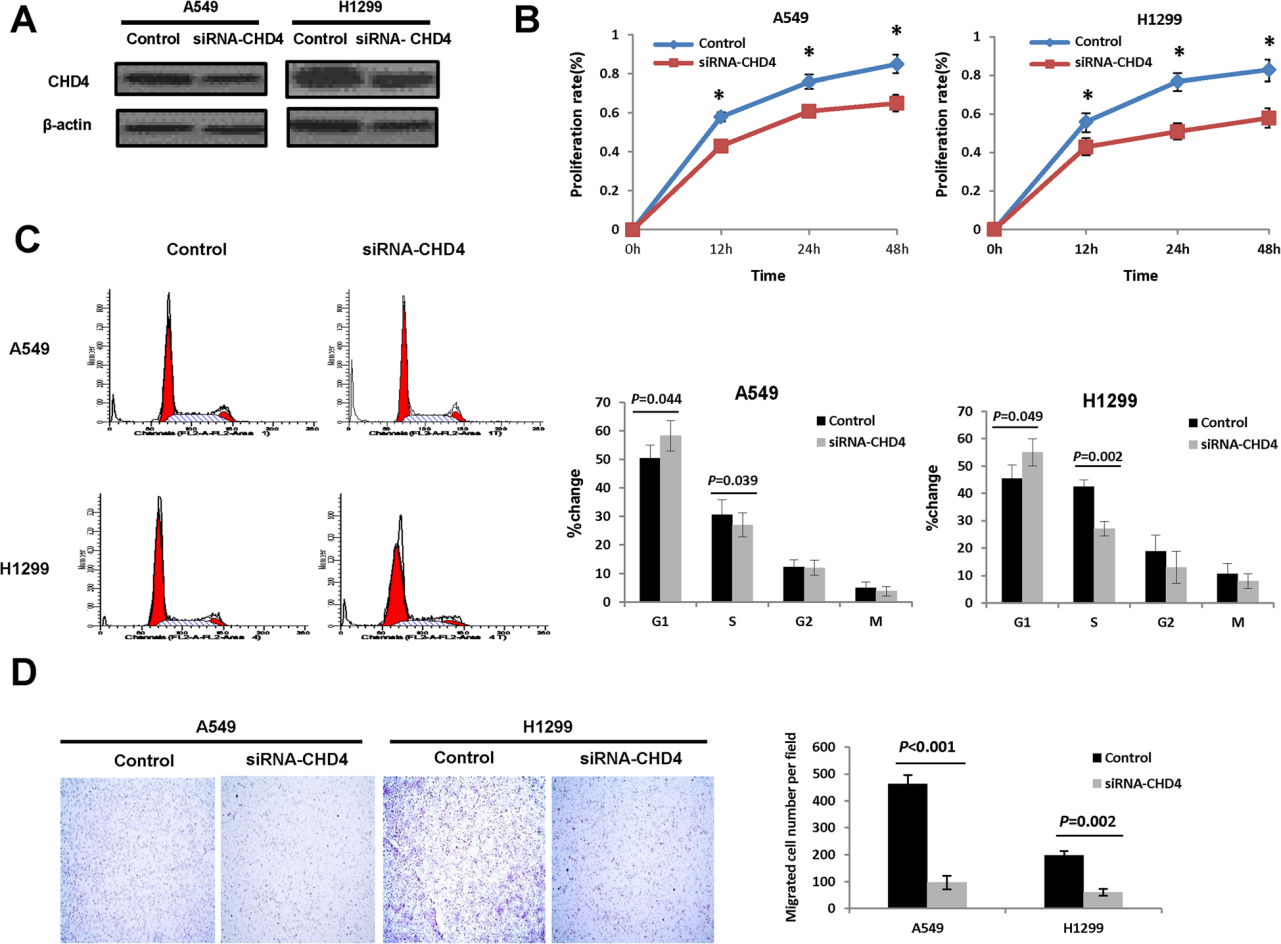
The effects of CHD4 down-regulation on NSCLC cell proliferation and migration. **a** Verification of siRNA-mediated knockdown of the CHD4 gene in A549 and H1299 cells by western blotting. Full-length blots were presented in Supplementary Fig. 2A. **b** The effects of siRNA-mediated knockdown of CHD4 on the proliferation of A459 and H1299 cells determined by MTT assay. **c** Representative results of the cell cycle analyses by FACS. CHD4 arrested the cell cycle in G1/S phase in A459 and H1299 cells (left). Quantification of the results of the cell cycle analysis was shown on the right. **d** Representative results of trans-well migration assays at 24 h after CHD4 knockdown in the A549 and H1299 cells. Statistical analysis of the differences between the groups was performed with an unpaired Student’s t-test. The results were shown as mean ± s.e.m., *, *P* < 0.05; **, *P* < 0.01; ***, *P* < 0.001

To further determine whether CHD4 represents a novel NSCLC-associated gene, we examined the roles of CHD4 in NSCLC development and progression. First, the expression levels of CHD4 in five NSCLC cell lines were determined by immunoblotting. Based on the immunoblotting results (Fig. S1), A549 and H1299 cells were selected for use in the CHD4 knockdown experiments, and successful knockdown by siRNA was confirmed by western blot analysis (Fig. 2a, Fig. S2A). This CHD4 knockdown was observed to markedly suppress the proliferation of A549 and H1299 cells (Fig. 2b). Consistently, the CHD4 knockdown was also observed to arrest the cell cycle at the G1/S phase (Fig. 2c). Using transwell assays (Fig. 2d), it was also shown that the reduced expression of CHD4 significantly inhibited cell migration. We therefore speculated that CHD4 might be a novel candidate tumor-associated gene in NSCLC.

### Up-regulation of CHD4 promotes NSCLC cell migration and proliferation in vitro

**Fig. 3:**
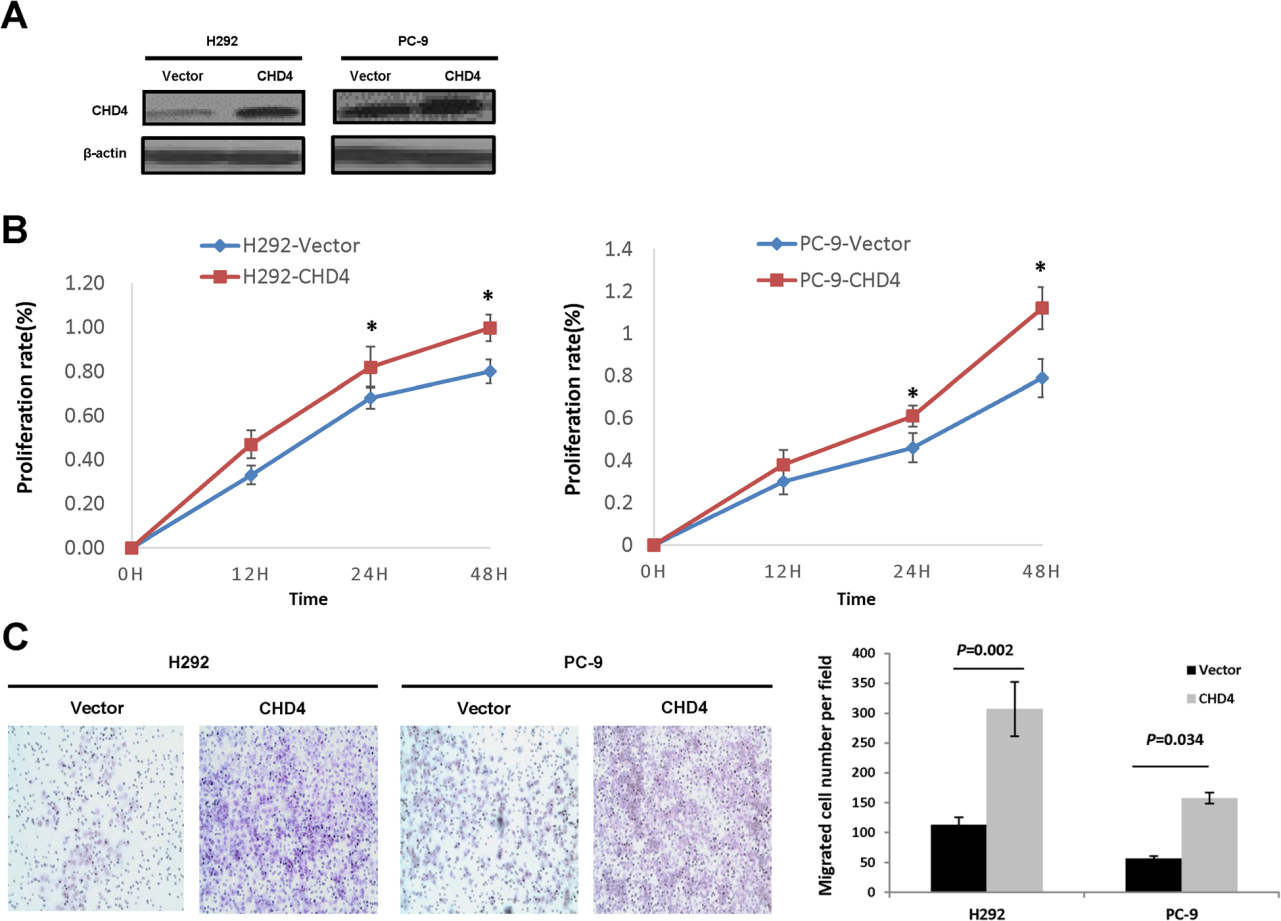
The effects of CHD4 up-regulation on NSCLC cell proliferation and migration. **a** Detection of over-expression of the CHD4 gene in H292 and PC-9 cells by western blotting analysis. Full-length blots were presented in Supplementary Fig. 2B. **b** The effects of over-expression of CHD4 on the proliferation of H292 and PC-9 cells by MTT assay. **c**. Representative results of transwell migration assays in CHD4-overexpressing H292 and PC-9 cells at 24 h. The results were shown as mean ± s.e.m., *, *P* < 0.05; **, *P* < 0.01; ***, *P* < 0.001

We then stably overexpressed CHD4 in H292 and PC-9 cell lines, which were confirmed by western blot analysis (Fig. 3a, Fig. S2B). Elevation of CHD4 expression could promote the proliferation rate at 24 and 48 h (Fig. 3b). By use of transwell assays, the overexpression of CHD4 was found to significantly increase the migratory potentials of the NSCLC cells (Fig. 3c). Taken together, these data demonstrated that increased expression of CHD4 plays an important role in NSCLC progression.

### Down-regulation of CHD4 reduces NSCLC proliferation in vivo

**Fig. 4:**
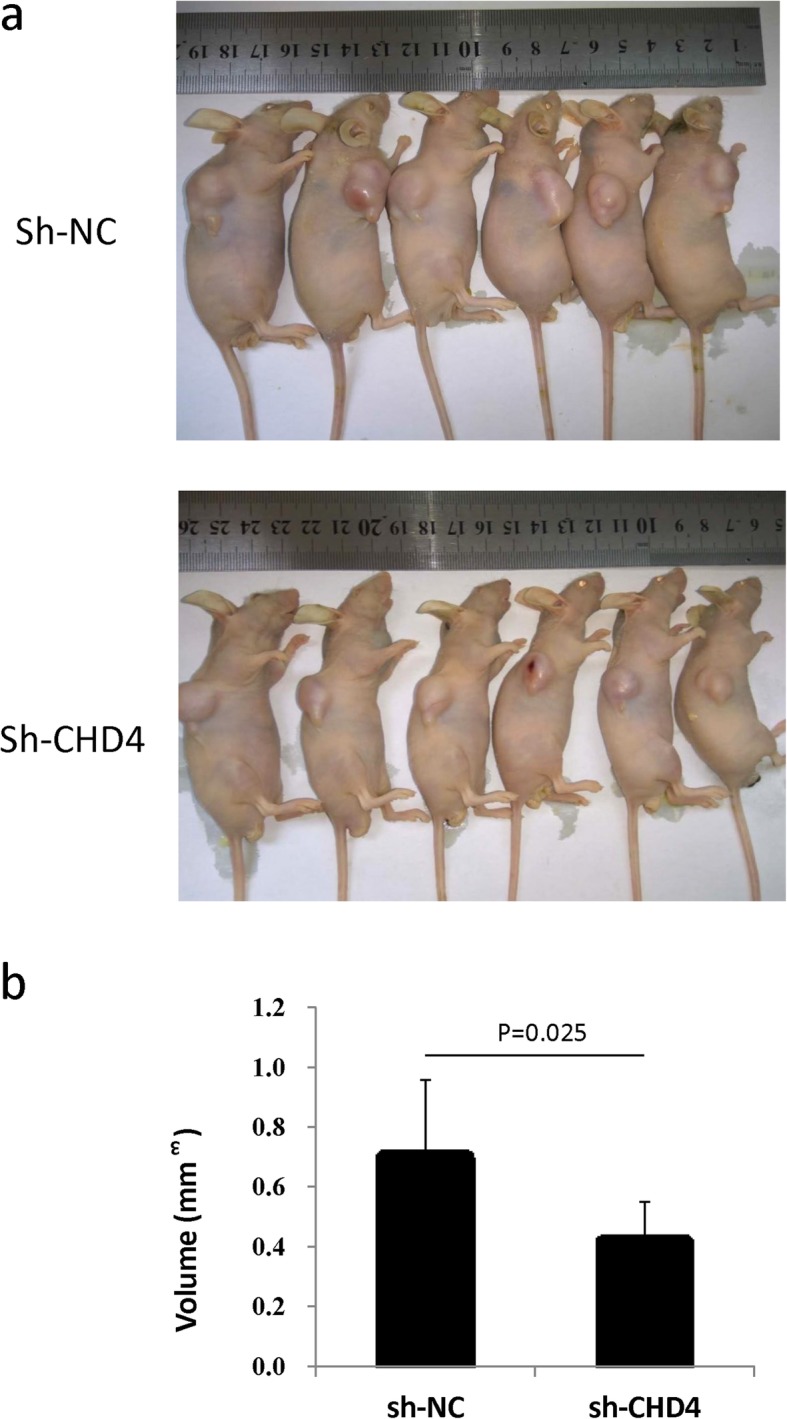
The effects of CHD4 on NSCLC cell tumor formation in nude mice. **a** Visual inspection of tumors in the two groups (Sh-NC and Sh-CHD4) of mouse models. **b** Volume of tumors of the two groups (Sh-NC and Sh-CHD4) of mouse models (*n* = 6). The results were shown as the mean ± S.D.; *P* < 0.05 was considered significant

We then stably suppressed CHD4 expression in the A549 cell line, and these CHD4-down-regulated A549 cells were injected subcutaneously into nude mice to investigate the effects of CHD4 on tumorigenicity. Following the in vivo analysis of tumorigenesis in nude mice, the growth properties of tumors were observed. It was shown that CHD4-down-regulated A549 cells formed much smaller (*P* < 0.05) tumors than A549-NC cells (Fig. 4a) (*n* = 6/group). Tumor volume was much decreased (*P* = 0.025) in the CHD4-down-regulated A549 cells (Fig. 4b), suggesting that CHD4 suppression could significantly decrease tumorigenicity in nude mice.

### CHD4 promotes NSCLC cell migration and proliferation by mediating the RhoA/ROCK signaling pathway

Given that CHD4 is overexpressed in NSCLC and functions as a novel tumor-promoting gene in NSCLC, we further sought to determine the mechanisms underlying CHD4-mediated promotion of NSCLC cell migration and proliferation. To define the cellular pathways in which CHD4 is involved, Gene Ontology (GO) functional analysis was performed. Overall, the analysis showed enrichment for cancer-dominant functions, such as DNA replication/messenger RNA (mRNA) processing, cell cycle/cell proliferation, and regulation of cytoskeleton, signaling by Rho GTPases pathway was included (Fig. S3), suggesting that these pathways may have important roles in NSCLC pathogenesis.

**Fig. 5:**
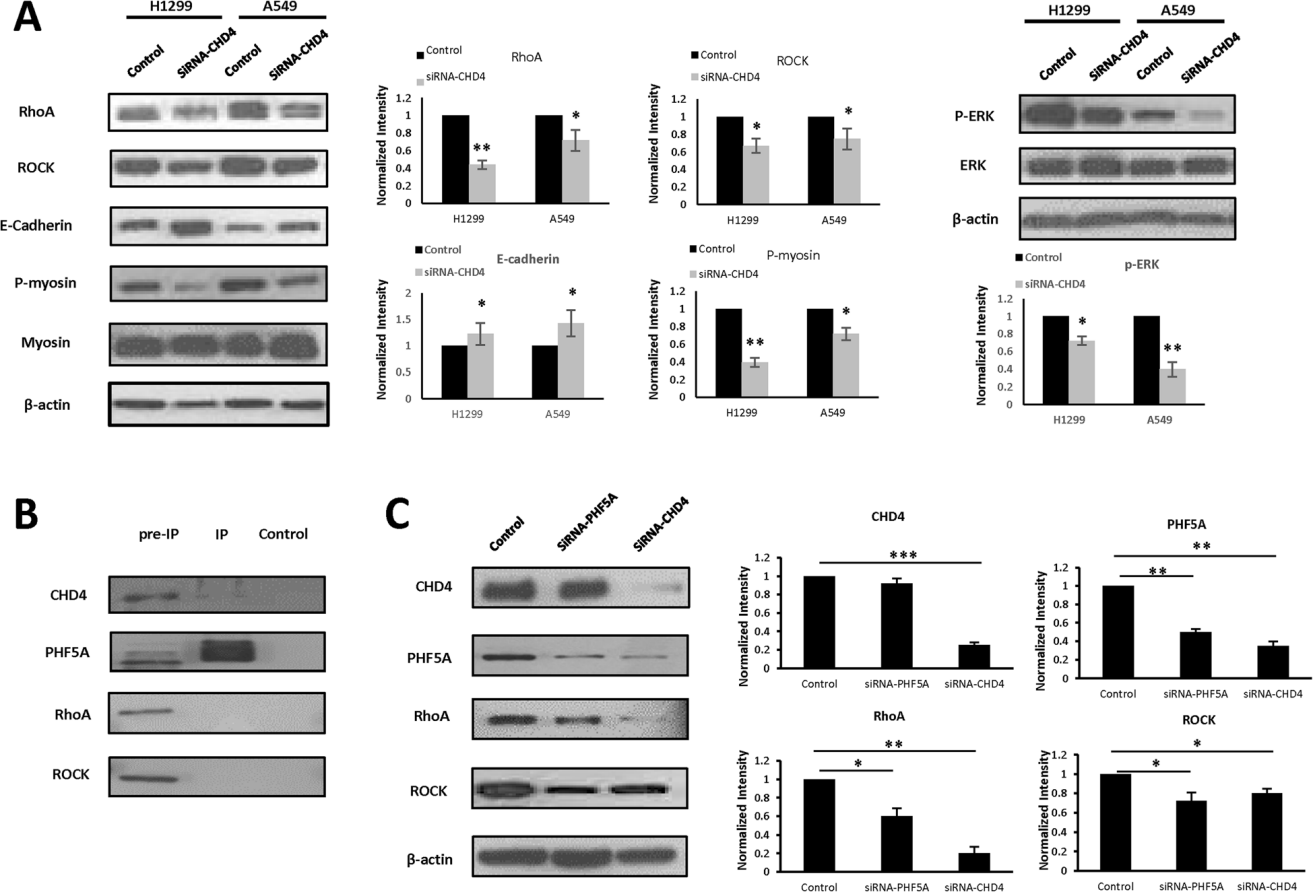
CHD4 down-regulation suppressed the RhoA/ROCK signaling pathway by interacting with PHF5A. **a** CHD4 down-regulation decreased RhoA, ROCK, p-myosin and p-ERK, but increased E-cadherin expression in both H1299 and A549 cell lines. (left and up), and the results of which were further quantified (right and down). β-actin served as a loading control. Full-length blots were presented in Supplementary Fig. 4. **b** The co-immunoprecipitation (IP) analysis of the CHD4 and PHF5A association in human A549 cells. Full-length blots were presented in Supplementary Fig. 5. **c** CHD4 suppression decreased the expression of PHF5A, while down-regulation of PHF5A could reduce RhoA, ROCK, rather than CHD4 expression in A549 cells (left), and the results of which was further quantified (right). β-actin served as a loading control. Full-length blots were presented in Supplementary Fig. 6 The results were shown as the mean ± S.D.; *P* < 0.05 was considered significant

According to the results of the GO functional analysis, CHD4 is likely associated with the RhoA/ROCK signaling pathway. To further explore the potential interaction of CHD4 with the RhoA/ROCK signaling pathway, we used CHD4-down-regulated cells to examine the RhoA/ROCK pathway. The results revealed that when CHD4 was suppressed, the expression levels of ROCK and RhoA and the downstream factors phospho-myosin were greatly reduced, while E-cadherin expression was elevated. Interestingly, we found that CHD4 knockdown could also reduce the expression of p-ERK, rather than ERK (Fig. 5a, Fig. S4). Together, these results suggested that CHD4 may promote NSCLC cell migration and proliferation via the RhoA/ROCK signaling pathway.

### CHD4 associates with the RhoA/ROCK pathway by regulation of PHF5A

We subsequently determined which factors participate in the regulation of the RhoA/ROCK pathway by CHD4. The GO functional analysis indicated that PHF5A, was likely associated with CHD4. To verify the binding of CHD4 to PHF5A, Co-IP was performed using A549 whole-cell lysates with antibodies against CHD4 and PHF5A, which identified an interaction between CHD4 and PHF5A (Fig. 5b, Fig. S5). These results suggest that PHF5A could bind to CHD4 and that it may participate in the RhoA/ROCK signaling pathway.

To identify whether PHF5A could mediate the RhoA/ROCK signaling pathway, siRNA against PHF5A was used to suppress the expression of PHF5A in A549 cells, and the expression levels of the downstream RhoA/ROCK signaling factors were determined. PHF5A down-regulation was found to reduce the expression levels of ROCK and RhoA (Fig. 5c, Fig. S6), indicating that PHF5A participates in NSCLC cell proliferation and metastasis through regulation of the RhoA/ROCK signaling pathway.

## Discussion

CHD4 is an ATP-dependent chromatin-remodeling protein that is a major subunit of the NuRD complex. It controls cell cycle progression and facilitates cell differentiation [16, 17]. CHD4 is essential in the DDR and has been linked to various oncogenic effects, including inducing abnormal stem cell renewal, suppressed differentiation, and altered cell-cycle control [18], suggesting that CHD4 plays an essential role in cancer development. In colorectal cancer, high CHD4 correlates with early disease recurrence and decreased overall survival [19]. The relevance of CHD4 to cancer development and progression was substantiated by our study; when comparing the expression levels of CHD4 in paired tumor and tumor-adjacent tissues, we found that CHD4 was more highly expressed in tumor tissues than in the tumor-adjacent tissues. Importantly, high CHD4 expression was strongly associated with aggressive tumor behavior and poor overall survival of NSCLC patients, indicating that CHD4 could be used as an independent factor for predicting NSCLC patient prognosis. Moreover, a prospective study is needed to further validate if CHD4 had a prediction value in overall survival of patients with NSCLC.

As mentioned in earlier studies, CHD4 acts as an important regulator of the G1/S cell-cycle transition by controlling p53 deacetylation [20]. CHD4 knockdown activates silenced TSGs, which represses colorectal cancer cell proliferation, invasion and metastasis [19]. However, in a study reported recently, TRPS1-CHD4/NuRD(MTA2) complex represses TP63 expression by involving decommission of TP63 enhancer, leading to a reduction of the ΔNp63 level and could reduce cell migration and invasion of breast cancer cells [7], which might lead to the resistance to CHD4 suppression. In the present study, we showed that, in NSCLC, CHD4 knockdown inhibited cell proliferative ability in vitro and in vivo, and led to cell cycle arrest at G1/S phase, while an increase of CHD4 promoted cell proliferative ability. Consistent with its effects in proliferation, CHD4 expression level was also correlated with the migrative potential of NSCLC cells. Further in vivo study using cell lines or patient-derived xenograft (PDX) models are needed to demonstrate the role of CHD4 in promoting migrative ability of NSCLC.

Several studies have attempted to elucidate the potential mechanisms of CHD4-mediated proliferation on cancer development. CHD4 and other components of the NuRD complex, which interacts with TWIST, could be recruited to the proximal regions of the E-cadherin promoter for transcriptional repression. Depletion of these components could efficiently suppress cell migration and invasion in cell culture and in lung metastasis in mice [21]. In CRC cells, CHD4 retention helps maintain DNA hypermethylation-associated transcriptional silencing. CHD4 knockdown alone reactivates the expression of E-cadherin and other genes; abnormal silencing of these genes potentially mediates escape from senescence, and proliferation, invasion and metastasis are therefore inhibited by CHD4 knockdown [19, 22]. In accordance with the aforementioned studies, the results of present study also demonstrated that CHD4 down-regulation promoted E-cadherin expression, which is important in the epithelial-to-mesenchymal transition. Furthermore, our GO functional analysis also found that CHD4 was associated with the RhoA/ROCK signaling pathway. By using western blotting, we confirmed that CHD4 down-regulation reduced RhoA, ROCK, phospho-myosin expression. These results illustrated that CHD4 could mediate cell motility and thus affect cancer cell metastasis.

Moreover, our results showed that p-ERK expression levels were attenuated with the suppression of CHD4, leading us to speculate that CHD4 may play a role in cancer cell proliferation via activation of the MAPK/ERK signaling pathway. In studies of pancreatic cancer, nuclear p-ERK staining levels were associated with poorer survival [23, 24] and this finding was in line with the correlation between CHD4 and survival observed in the current study.

Using GO functional analysis, we screened several factors to clarify which factors may be associated with CHD4. The results showed that CHD4 reduction suppressed PHF5A expression levels. PHF5A is a highly conserved PHD-zinc finger domain protein that facilitates interactions between the U2 snRNP complex and DNA/RNA helicases [25]. Additionally, PHF5A facilitates interactions with specific histone marks on chromatin-bound nucleosomes through its PHD domain [26–29]. PHF5A inhibition also compromised GSC tumor formation in vivo and inhibited the growth of established GBM patient-derived xenograft tumors [30]. It is also reported that PHF5A played an oncogenic role via AS in lung adenocarcinoma [31]. Our results demonstrated that PHF5A was mediated by CHD4 and then regulated the RhoA/ROCK pathway. Further studies with in vitro and in vivo experiments are needed to demonstrate the biological role of PHF5A in proliferation and migration of NSCLC.

## Conclusion

In summary, to the best of our knowledge, this study demonstrated that CHD4 could be an independent factor for NSCLC prognosis and could be used as a biomarker for identifying NSCLC risk stratification. The results indicated that it has a novel role in NSCLC migration and proliferation. Molecular studies revealed that the function of CHD4 in promoting cell migration and proliferation was via activation of the RhoA/ROCK signaling pathway by its interaction with PHF5A. Our findings suggested that CHD4 could be a good therapeutic target to consider for cancer management.

## Supplementary information

**Additional file 1: Figure S1.** Western blot analysis of CHD4 expression levels in NSCLC cell lines.

**Additional file 2: Figure S2.** (A) Full-length blots of western blot analysis of CHD4 expression levels in A549 and H1299 cells. (B) Full-length blots of western blot analysis of CHD4 expression levels in H292 and PC-9 cells. (left) cropping gels, (right) original, full-length blots. The orange lines indicated the corresponding bands of the cropping blots.

**Additional file 3: Figure S3.** GO functional analysis on significant related-genes induced by CHD4 down-regulation (genes with a fold increase ≥9 were included). The significant enrichment of signaling by Rho GTPases pathway was identified.

**Additional file 4: Figure S4.** Full-length blots of western blot analysis of RhoA, ROCK, p-myosin p-ERK and E-cadherin expression in both H1299 and A549 cell lines. (left) cropping blots, (right) original, full-length blots. The orange lines indicated the corresponding bands of the cropping blots.

**Additional file 5: Figure S5.** Full-length blots of IP analysis in A549 cells. (left) cropping blots, (right) original, full-length blots.

**Additional file 6: Figure S6.** Full-length blots of western blot analysis of CHD4, RhoA, ROCK and PHF5A expression in A549 cells. (left) cropping blots, (right) original, full-length blots.

**Additional file 7: Table S1.** Clinical profile and correlation between the clinicopathological features and CHD4 expression.

## Data Availability

The datasets used and/or analyzed during the current study are available from the corresponding author on reasonable request.

## Abbreviations

NSCLC: Non-small cell lung cancer
CHD4: Chromodomain helicase DNA-binding protein 4
ALK: Anaplastic lymphoma kinase
GO: Gene Ontology
TSGs: Tumor-suppressor genes
NuRD: Nucleosome remodeling deacetylase
DDR: DNA damage response
PI: Propidium iodide
mRNA: Messenger RNA
PHF5A: PHD finger protein 5A
