# Supplementary figures and images for: CHD4 mediates proliferation and migration of non-small cell lung cancer via the RhoA/ROCK pathway by regulating PHF5A
Source: BMC Cancer. 2020 Mar 30;20:262. doi: 10.1186/s12885-020-06762-z (PMC7106713; doi:10.1186/s12885-020-06762-z)

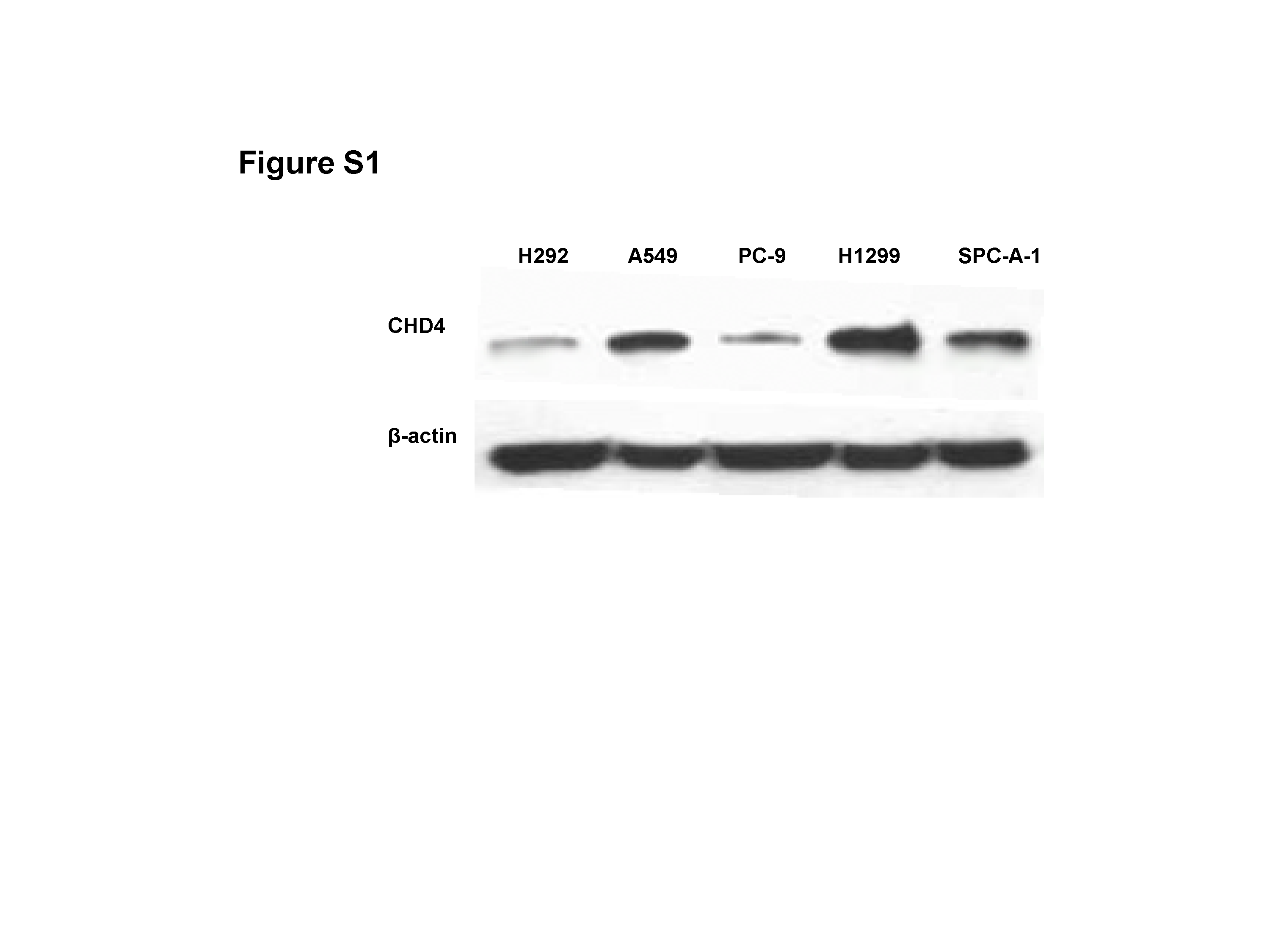

Supplement: Supplementary file 1 — Additional file 1: Figure S1. Western blot analysis of CHD4 expression levels in NSCLC cell lines. [file 12885_2020_6762_MOESM1_ESM.tiff]

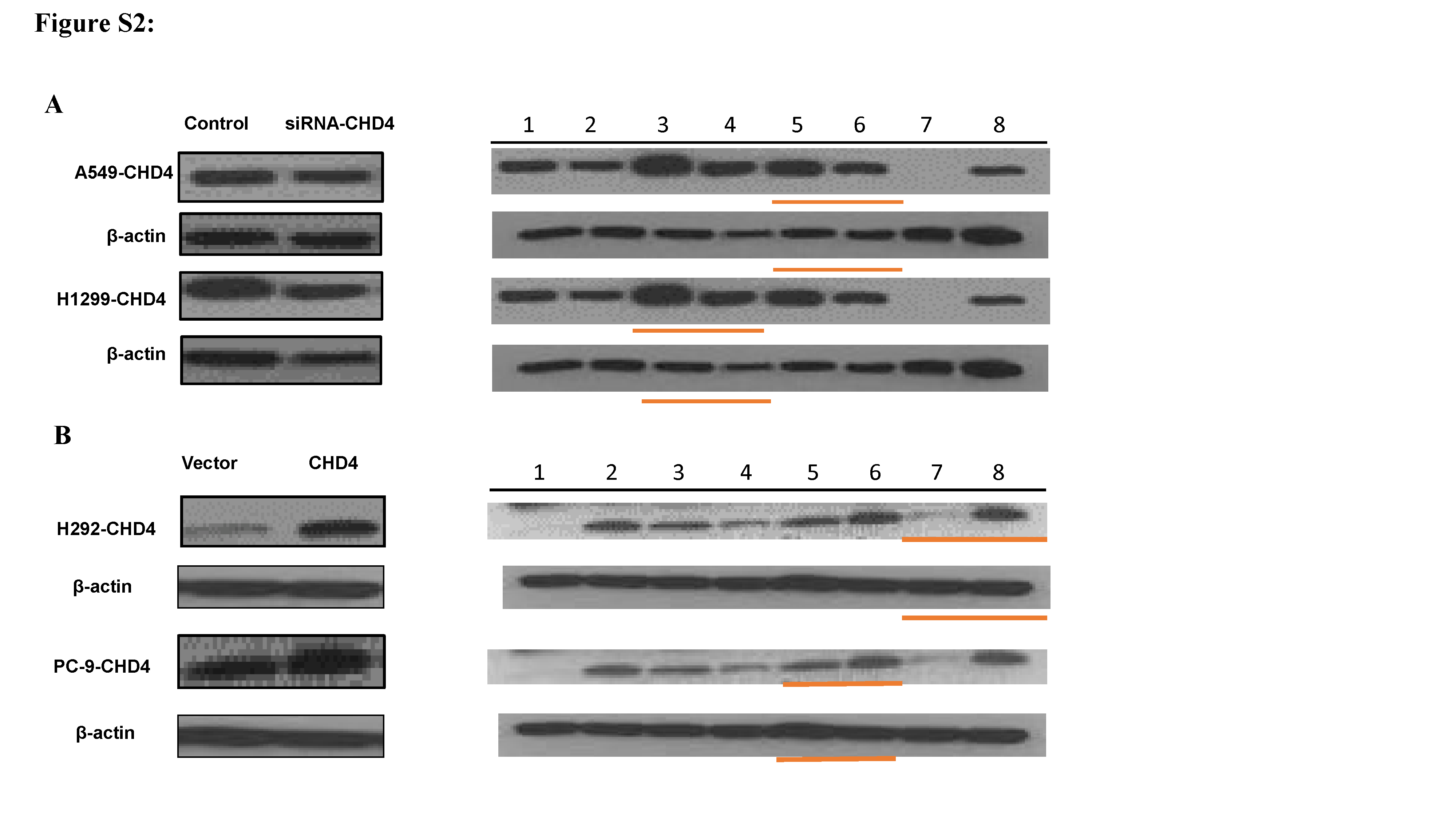

Supplement: Supplementary file 2 — Additional file 2: Figure S2. (A) Full-length blots of western blot analysis of CHD4 expression levels in A549 and H1299 cells. (B) Full-length blots of western blot analysis of CHD4 expression levels in H292 and PC-9 cells. (left) cropping gels, (right) original, full-length blots. The orange lines indicated the corresponding bands of the cropping blots. [file 12885_2020_6762_MOESM2_ESM.tiff]

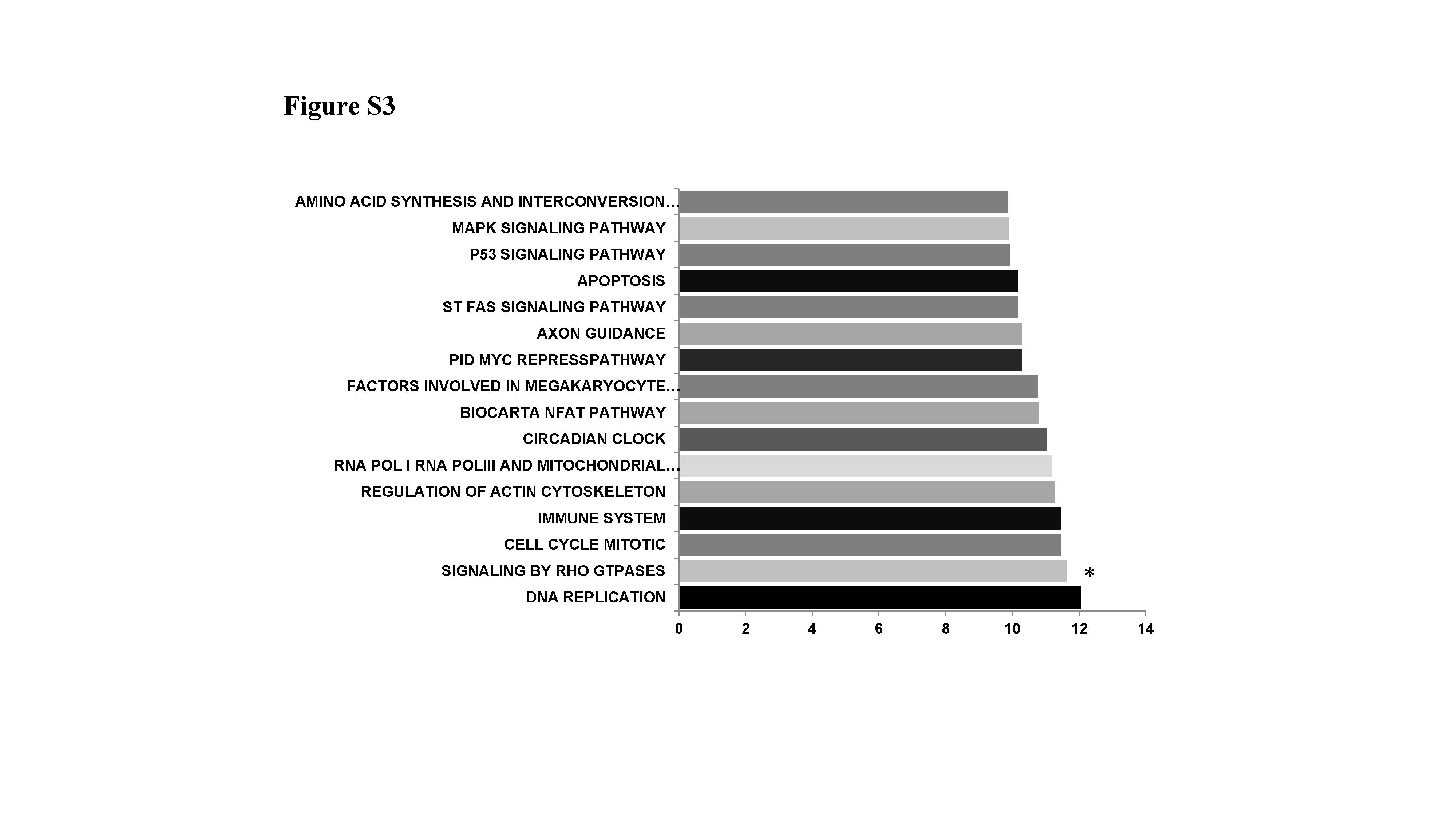

Supplement: Supplementary file 3 — Additional file 3: Figure S3. GO functional analysis on significant related-genes induced by CHD4 down-regulation (genes with a fold increase ≥9 were included). The significant enrichment of signaling by Rho GTPases pathway was identified. [file 12885_2020_6762_MOESM3_ESM.tiff]

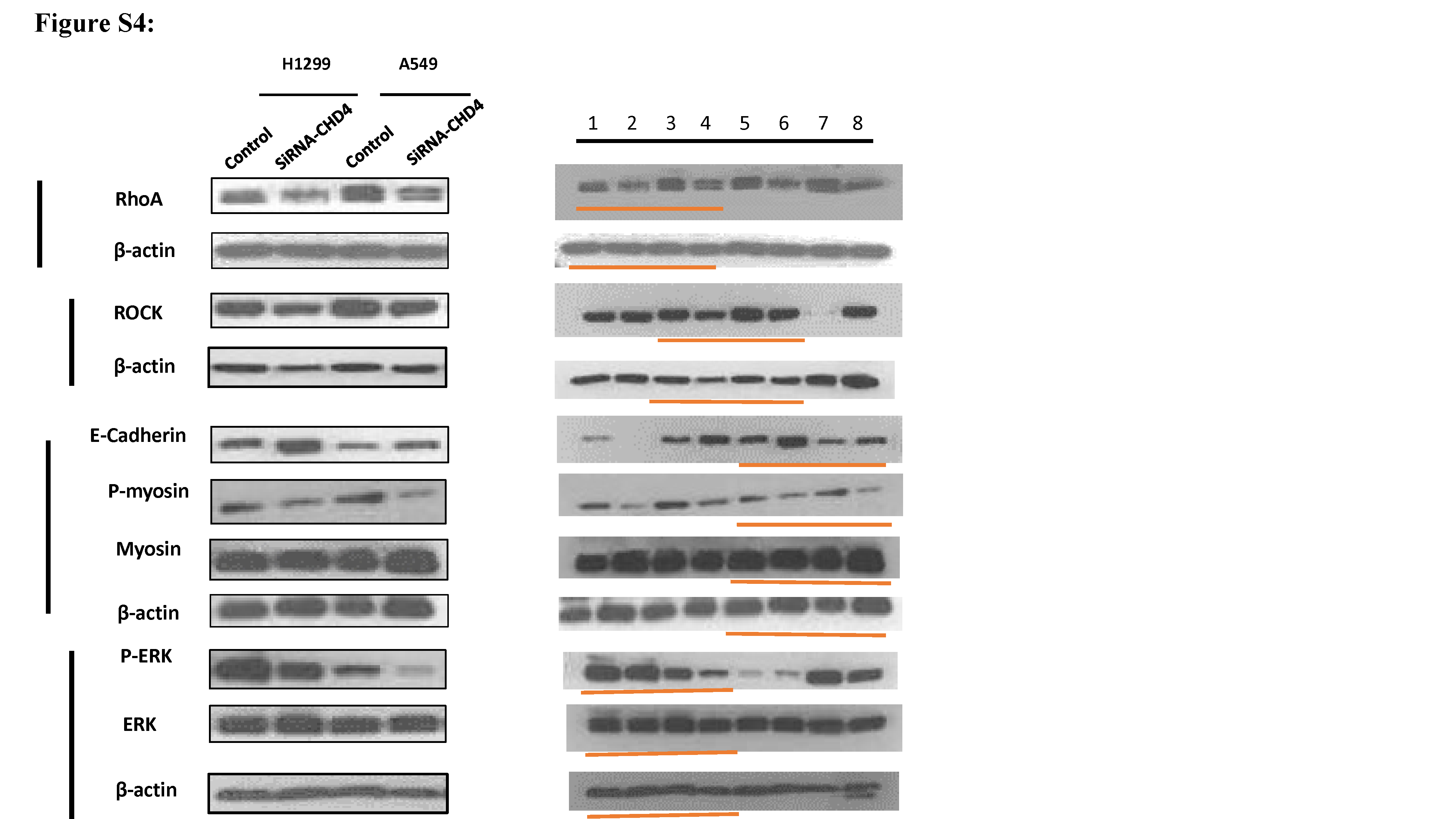

Supplement: Supplementary file 4 — Additional file 4: Figure S4. Full-length blots of western blot analysis of RhoA, ROCK, p-myosin p-ERK and E-cadherin expression in both H1299 and A549 cell lines. (left) cropping blots, (right) original, full-length blots. The orange lines indicated the corresponding bands of the cropping blots. [file 12885_2020_6762_MOESM4_ESM.tiff]

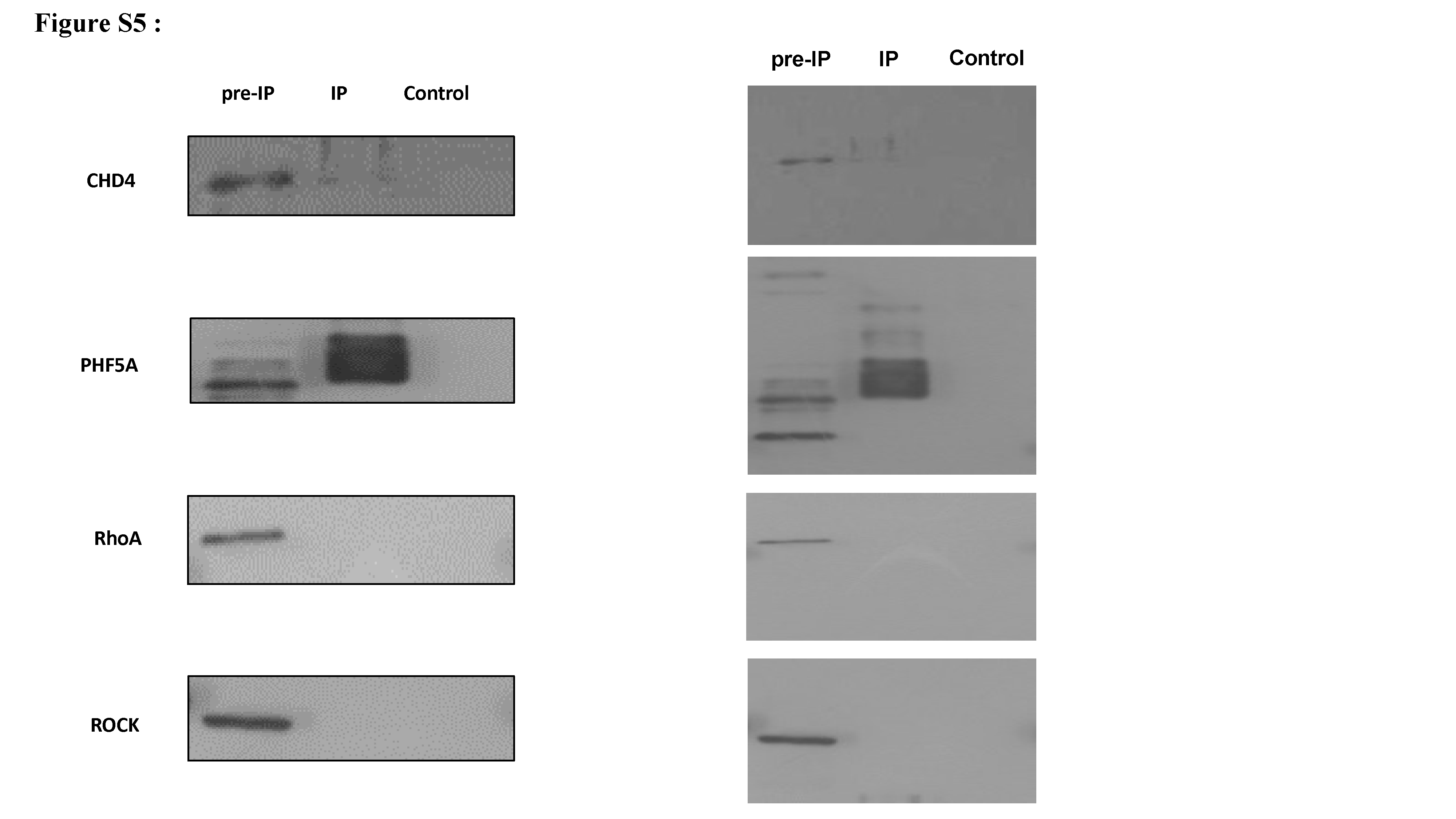

Supplement: Supplementary file 5 — Additional file 5: Figure S5. Full-length blots of IP analysis in A549 cells. (left) cropping blots, (right) original, full-length blots. [file 12885_2020_6762_MOESM5_ESM.tiff]

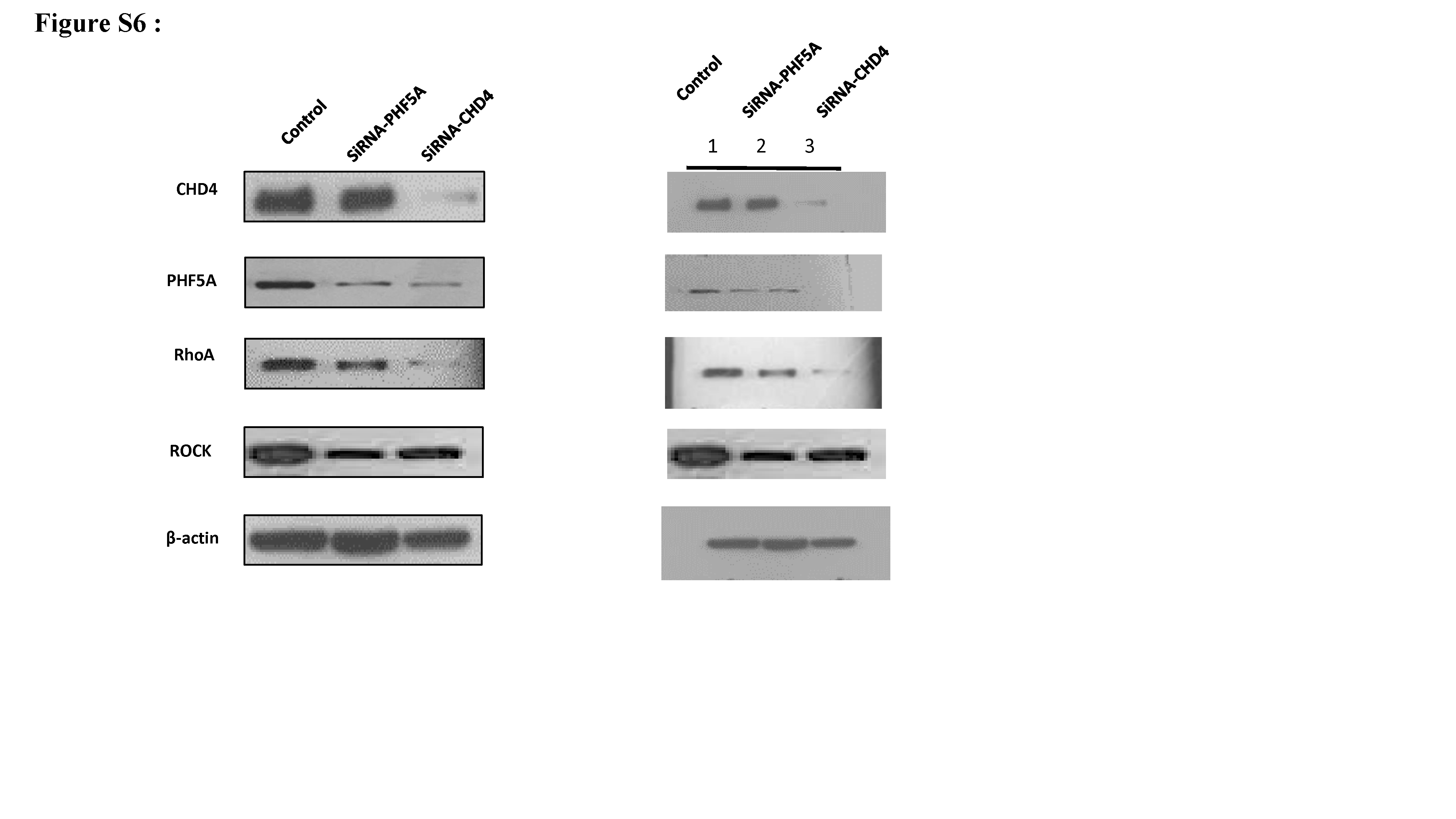

Supplement: Supplementary file 6 — Additional file 6: Figure S6. Full-length blots of western blot analysis of CHD4, RhoA, ROCK and PHF5A expression in A549 cells. (left) cropping blots, (right) original, full-length blots. [file 12885_2020_6762_MOESM6_ESM.tiff]
