# Supplementary material for: CHD4 mediates proliferation and migration of non-small cell lung cancer via the RhoA/ROCK pathway by regulating PHF5A
Source: BMC Cancer. 2020 Mar 30;20:262. doi: 10.1186/s12885-020-06762-z (PMC7106713; doi:10.1186/s12885-020-06762-z)
Supplement: Supplementary file 7 — Additional file 7: Table S1. Clinical profile and correlation between the clinicopathological features and CHD4 expression. [file 12885_2020_6762_MOESM7_ESM.docx]

***TableS1:*** *Clinical profile and correlation between the clinicopathological features and expression of CHD4.*

| *VARIABLE* | *No. of patients* | | *CHD4 expression* | | |  | *P-value* | |
| --- | --- | --- | --- | --- | --- | --- | --- | --- |
|  |  | | *Negative/Weak* | | *Strong* | | |  |
| *Age(years)*  0.088 | | | | | | | | |
| ≤55 | 29 | | 9 | 20 | | | |  |
| >55 | 117 | | 57 | 60 | | | |  |
| *Gender* |  | |  |  | | | | 0.102 |
| Male | 107 | | 44 | 63 | | | |  |
| Female | 39 | | 22 | 17 | | | |  |
| *Smoking status* | |  |  |  | | | |  |
| Yes | | 87 |  |  | | | |  |
| No | | 59 |  |  | | | |  |
| *Histology*  Adenocarcinoma  Squamous cell carcinoma | | 80  66 | 41  25 | 39  41 | | | | 0.108 |
| *Differentiation* |  | |  |  | | | | 0.241 |
| Well and moderately | 75 | | 38 | 37 | | | |  |
| Poorly | 71 | | 28 | 43 | | | |  |
| *TNM Stage* |  | |  |  | | | | 0.001* |
| I, II | 97 | | 53 | 44 | | | |  |
| III, IV | 49 | | 13 | 36 | | | |  |
| *Tumor stage* |  | |  |  | | | | 0.002* |
| T1 and T2 | 111 | | 58 | 53 | | | |  |
| T3 and T4 | 35 | | 8 | 27 | | | |  |
| *Lymph node metastasis* |  | |  |  | | | | 0.005* |
| No | 88 | | 48 | 40 | | | |  |
| Yes | 58 | | 18 | 40 | | | |  |

Abbreviation: No., number; TNM, tumor node metastasis.

*, significant.
